# Halophytic *C*‑Glycosyltransferases Enable *C*‑Glycosylation in Organic Solvents

**DOI:** 10.1021/acsomega.5c07452

**Published:** 2025-11-16

**Authors:** Onur Kırtel, Lea Helena Strother, Natalia Putkaradze, Ditte Hededam Welner

**Affiliations:** The Novo Nordisk Foundation Center for Biosustainability, 5205 Technical University of Denmark, Søltofts Plads 220, Lyngby DK-2800, Denmark

## Abstract

*C*-glycosyltransferases from glycosyltransferase family 1 transfer sugar moieties to carbon atoms in the substituted aromatic rings of various small molecules. They are coveted biocatalysts for the synthesis of high-value glycosides since the resulting β-*C*-glycosidic linkage is usually more stable *in vivo* and *in vitro* than its *O*-glycosidic counterpart. One of the main bottlenecks in the biocatalytic glycosylation processes of small molecules is the low aqueous solubility of the acceptor substrate, drastically limiting product yields. One solution is to conduct the reaction in organic solvents provided the enzyme activity is preserved. Salt-tolerant organisms often have enzymes that are tolerant of organic solvents. In this work, we report the discovery and characterization of three novel *C*-glycosyltransferases from halophytes (i.e., salt-tolerant plants) through sequence mining. All enzymes converted phloretin to its *C*-glucosides efficiently with high regioselectivity and surprisingly exhibited significantly enhanced conversion yields in the presence of acetonitrile or methanol—up to 1563% for the enzyme from*Trifolium fragiferum* (TfCGT) in 30% methanol (v/v). The halophytic *C*-glycosyltransferases had activity maxima at 55–65 °C and pH 8.7–10.0. They exhibited varying chemostability profiles toward their substrate, with the newly described enzyme from *Phragmites australis* (PaCGT) performing remarkably well under low enzyme and high phloretin conditions. In line with the extreme adaptations of their hosts, halophytic *C*-glycosyltransferases might have evolved to perform better in water-restricted conditions (e.g., in highly saline or arid habitats), thus holding great potential for industrial glycosylation processes with reduced enzyme and increased aglycon concentrations.

## Graphical abstract

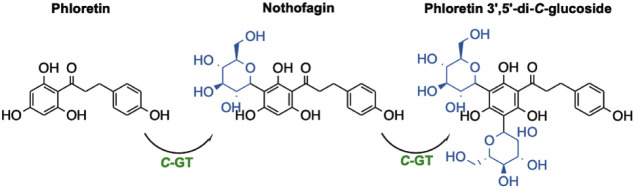

## 1 Introduction

Enzymatic glycosylation offers facile and regioselective modification of natural products, enabling a straightforward way to improve the solubility/stability or reduce the toxicity of small molecules among other functions. Glycosyltransferase family 1 (GT1) enzymes (also known as uridine diphosphate-dependent glycosyltransferases, UGTs) from plants are the first line of choice when it comes to glycosylation of natural products due to their remarkable taxonomic diversity and a wide array of acceptor specificities. As of June 2025, the GT1 sequence space is comprised of more than 66,000 enzymes according to the CAZy database (http://www.cazy.org/),^1^ with 80 crystal structures available. Within GT1, *C*-glycosyltransferases (*C*-GTs) are unique in linking a sugar moiety with a natural product through β-*C*-glycosidic bonds,^2,3^ and they were described for the first time in the early 2000s.^4–6^ The resulting *C*-glycosides carry substantial biotechnological potential^7–11^ and have high chemical and metabolic stability.^12,13^ Nevertheless, one of the major issues that hinder industrial-scale enzymatic *C*-glycosylation of natural products is the very low aqueous solubility of the aglycons.^14–16^ To meet industrial requirements for enzymatic *C*-glycosylation, the discovery of novel *C*-GTs with improved compatibility with different media is required.

Polar organic solvents can render aglycons more soluble by orders of magnitude.^17^ However, the majority of characterized GT1 enzymes are from mesophilic organisms (https://www.cazy.org/GT1_characterized.html)^1^ and therefore are expected to be severely hampered in water-restricted milieu. In contrast, enzymes from halophilic/halotolerant organisms can potentially retain their activity in the presence of organic solvents due to the similarly reduced water availability in high-salinity conditions.^18,19^ Halophytes are plants that thrive in saline environments, whether coastal habitats, salt marshes, or salt deserts.^20^ They achieve this through various mechanisms, namely, the excretion of salt through salt bladders, alterations in membrane structures, regulation of cellular ion homeostasis, and the detoxification of reactive oxygen species,^21^ the latter being achieved through secondary metabolites such as flavonoids.^22^ Halophytes are a rich source of a wide range of bioactive phytochemicals and likely GT1 enzymes, although no systematic study has addressed this.

Although the salt (and organic solvent) tolerance of halophytic enzymes cannot be generalized and is highly dependent on the subcellular localization of the proteins,^23,24^ they still represent an intriguing and underexplored group of enzymes with substantial potential in industrial activities. In this work, we have identified three phloretin *C*-GTs from three distinct halophytes, namely, PaCGT from *Phragmites australis* (common reed), PdCGT from *Phoenix dactylifera* (date palm), and TfCGT from *Trifolium fragiferum* (strawberry clover). All enzymes glycosylated phloretin to its mono- or di-*C*-glucosides, namely, nothofagin (phloretin 3-*C*-glucoside) and phloretin 3′,5′-di-*C*-glucoside, with PaCGT and TfCGT exhibiting perfect regioselectivity with exclusive synthesis of these two glucosides, respectively. Most importantly, halophytic *C*-GTs not only tolerated the presence of organic solvents, methanol and acetonitrile, but also demonstrated a striking increase in phloretin conversion yields, up to 1500% in the case of TfCGT, with 9 times less enzyme than that employed in aqueous buffers. This enhancement was not observed with nonhalophytic *C*-GTs tested. PaCGT showed remarkable chemostability toward the aglycon even at low enzyme concentrations, in contrast to many known GT1 enzymes,^25^ as well as superior regioselectivity for nothofagin. These findings represent a first glimpse into an enzyme class of high biotechnological potential and open possibilities for low-impact industrial *C*-glycosylation of natural products.

## 2 Results

### 2.1 Sequence Mining for Halophytic *C*-GTs

To discover novel *C*-GTs with potential for application in organic solvents, we carried out sequence mining targeted toward halophytic plant genomes, using five well-characterized *C*-GTs from *Trollius chinensis* (6JTD),^26^ *Glycyrrhiza glabra* (6L5S),^27^ *Citrus japonica* (BBA18062.1),^28^ *Mangifera indica* (7VAA),^29^ and *Oryza sativa* Indica group (C3W7B0.1)^30^ as queries for BLAST (tblastn).^31^ The BLAST searches were restricted to taxonomic familia that are known to contain halophytic species (see Supporting Information for the familia included). The family names were taken from the eHALOPH database (https://ehaloph.uc.pt) on Aug. 25, 2023. The number of hits with >50% sequence identity to each query sequence varied between 28 and 136, and these were further filtered according to their halophytic origin (the familia mentioned above contain both halophytic and nonhalophytic species), presence of the DPF­(FL)^2^ and the Plant Secondary Product Glycosylation^32^ motifs, instability index <45 as predicted by ExPASy ProtParam,^33^ and at least 300 mM salt concentration tolerated by the origin species according to the eHALOPH database. This filtering process brought down the total number of hits to a mere six, out of which four belonged to *Phoenix dactylifera* and two belonged to *Phragmites australis* and *Trifolium fragiferum* each. We chose one sequence from each species, resulting in three putative *C*-GTs from three plant species with differing lifestyles (Table 1). An overview of the sequences evaluated and chosen can be seen in the phylogenetic tree in Figure 1. *Phoenix dactylifera* (date palm) and *Phragmites australis* (common reed) are classified as hydrohalophytes,^34,35^ with the former found in the tropical and subtropical habitats of Asia and Africa,^36^ while the latter is an invasive species that easily expands into salt marshes. *Trifolium fragiferum* (strawberry clover) is a coastal species that is tolerant to moderate soil salinity.^37^ The previously described GgCGT^27^ from the halophyte *Glycyrrhiza glabra* (licorice), which can tolerate up to 800 mM NaCl,^38^ was chosen as the positive control in the experiments since this is a well-characterized enzyme with phloretin *C*-glycosylation activity reported.^27^ Phloretin was chosen as the acceptor substrate for *C*-GT activity screening since it is one of the most used acceptor molecules in *C*-glycosylation studies^3^ and it contains multiple *C*- and *O*-glycosylation sites, thus enabling the assessment of enzyme regioselectivity and *O*-/*C*-functionality.

**Table 1:** General Information on the *C*-GT Enzymes Used in This Study

Enzyme name	Accession number	Origin species	Plant type	Maximum salinity tolerated by plant	Reference
MiCGT	7VA8	*Mangifera indica*	Nonhalophyte	88 mM^39^	^40^
FcCGT	BBA18062.1	*Fortunella crassifolia*	Nonhalophyte	No data	^28^
VaCGT	XP_017438553.1	*Vigna angulariz*	Glycophyte	195 mM	^41^
GgCGT	6L5S	*Glycyrrhiza glabra*	Halophyte	800 mM	^27^
PdCGT	XP_038986326.1	*Phoenix dactylifera*	Hydrohalophyte	300 mM	This study
PaCGT	XP_062228688.1	*Phragmites australis*	Hydrohalophyte	500 mM	This study
TfCGT	OX940789.1^a^	*Trifolium fragiferum*	Halophyte	350 mM^b^	This study

a Genome assembly.

b Optimum salinity: 160 mM.

**Figure 1:**
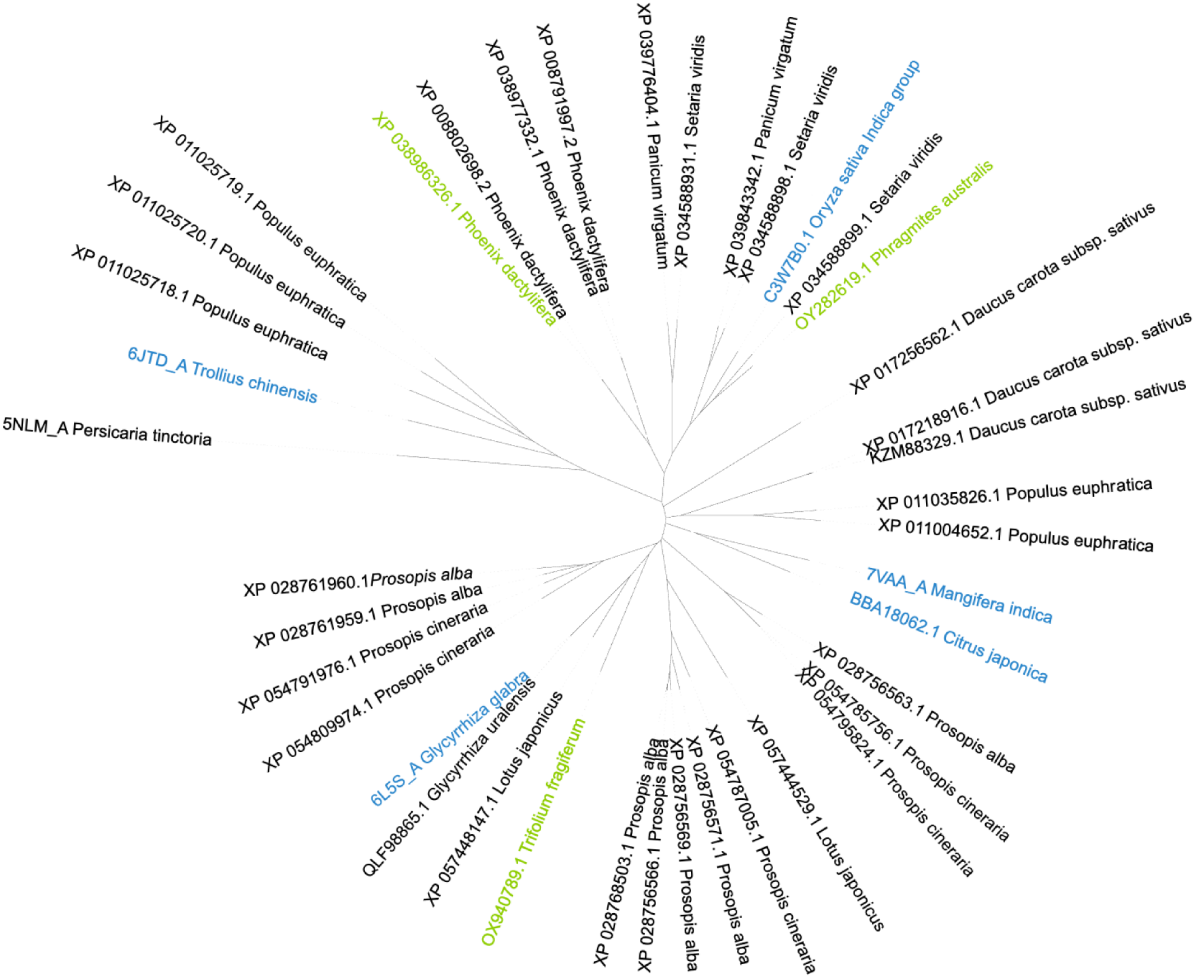
Phylogenetic tree showing the distribution of the query (blue) and the selected (green) halophytic *C*-GT sequences. Sequences that were left out during the filtering process are shown in black. The sequence 5NLM_A (*Persicaria tinctoria* GT1) was used as the outgroup.

### 2.2 Enzyme Production and Glycosylation Screening

Although the heterologous expression of the enzymes in *Escherichia coli* resulted in either low production titers (usually less than 1 mg of enzyme from 1 L of culture after immobilized metal affinity chromatography) or low solubility as determined by SDS-PAGE analysis, the amounts were sufficient for the required experiments. SDS-PAGE gel image analysis of the enzyme fractions used in the assays can be found in Supporting Information 2, while the HPLC standard curves for phloretin and its two glycosides are given in Supporting Information 3. All three enzymes, namely, PaCGT, PdCGT, and TfCGT, were found to be active on phloretin with varying product specificities (Figure 2). The initial glycosylation tests were run with 50 μg/mL total protein and 50 μM phloretin with an excess of 5 mM UDP-Glc. PdCGT converted 100% of phloretin to predominantly nothofagin and some phloretin 3′,5′-di-*C*-glucoside in 1 h, and the overnight reaction resulted in further glycosylation of the former to the latter with a final product ratio of 24% nothofagin and 76% phloretin 3′,5′-di-*C*-glucoside (Figure 2c). PaCGT and TfCGT were superior in terms of regioselectivity: They converted 100% of phloretin to exclusively nothofagin or phloretin 3′,5′-di-*C*-glucoside in 1 h, respectively (Figure 2b and d). It is worth pointing out that the remarkable regioselectivity of PaCGT–nothofagin was not further glycosylated to phloretin 3′,5′-di-*C*-glucoside even at the end of an overnight reaction. The positive control, GgCGT, converted almost all phloretin to a mixture of nothofagin (66%) and phloretin 3′,5′-di-*C*-glucoside (34%) in 10 min, with complete glycosylation of nothofagin to the latter in 1 h (Figure 2a). A similar result was reported for GgCGT before, where all phloretin was converted to nothofagin in 10 min, followed by complete further glycosylation to phloretin 3′,5′-di-*C*-glucoside in 35 min.^27^ An interesting phenomenon was observed in the GgCGT reaction when a significant portion of phloretin was converted to nothofagin even in the *t* = 0 sample, where an equal volume of 100% methanol was immediately added to the mixture to stop the reaction. To verify that this was not caused by a high reaction rate, we first added methanol to the reaction mixture, followed by the enzyme. Still there was almost 40% conversion to nothofagin and ca. 1% phloretin 3′,5′-di-*C*-glucoside in the presence of 50% methanol (v/v) in a tube on ice (*t* = 0 spectrum in Figure 2a). This inspired us to investigate the effect of polar organic solvents on the *C*-GT activity.

**Figure 2:**
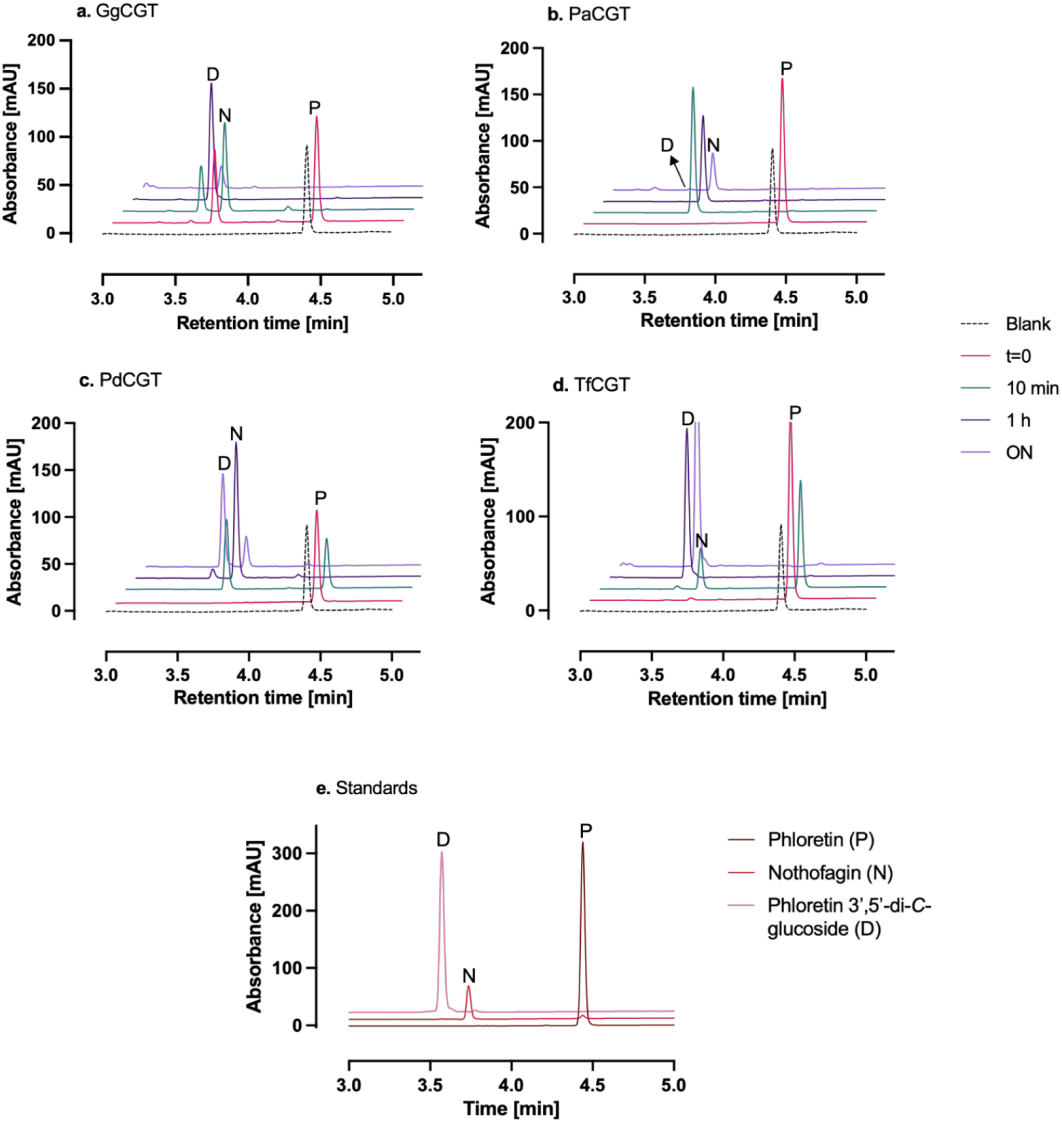
HPLC chromatograms showing the product specificities of halophytic *C*-GTs with phloretin. Reactions were run at 30 °C and contained 50 μg/mL total protein and 50 μM phloretin. “Blank” indicates a reaction mixture without any enzyme. P: phloretin (RT: 4.440 min); N: nothofagin (RT: 3.737 min); D: phloretin 3′,5′-di-*C*-glucoside (RT: 3.570 min); ON: overnight. In ON reactions with GgCGT and PaCGT, the areas of the D and N peaks, respectively, were reduced, possibly due to further glycosylation.

### 2.3 Glycosylation in Organic Solvents

Surprisingly, the four halophytic *C*-GTs not only tolerated the presence of organic solvents, but also in most cases, their activity was markedly enhanced when reactions were run in media containing acetonitrile or methanol (Figure 3). 30% acetonitrile (v/v) was the least productive solvent and detrimental to enzyme activity in all cases except for GgCGT, where the conversion remarkably reached 665% of that of the reaction in aqueous buffer with no solvent added (Figure 3a). Strikingly, after a 10-min reaction, GgCGT in 15% acetonitrile (v/v) converted an order of magnitude (1095%) more phloretin than in purely aqueous solution. A similar trend was seen for PdCGT and PaCGT, where maximum relative conversion yields (412% and 228%, respectively) were observed in reactions with 15% acetonitrile (v/v) (Figure 3b,c). For TfCGT, 30% methanol (v/v) provided the highest relative conversion yields, a striking 1563% (Figure 3d). As the concentrations of phloretin and its glucosides were low (50 μM initial phloretin), the choice of quench solvent (methanol versus acetonitrile) is not expected to have a measurable impact on the reported yields.

**Figure 3:**
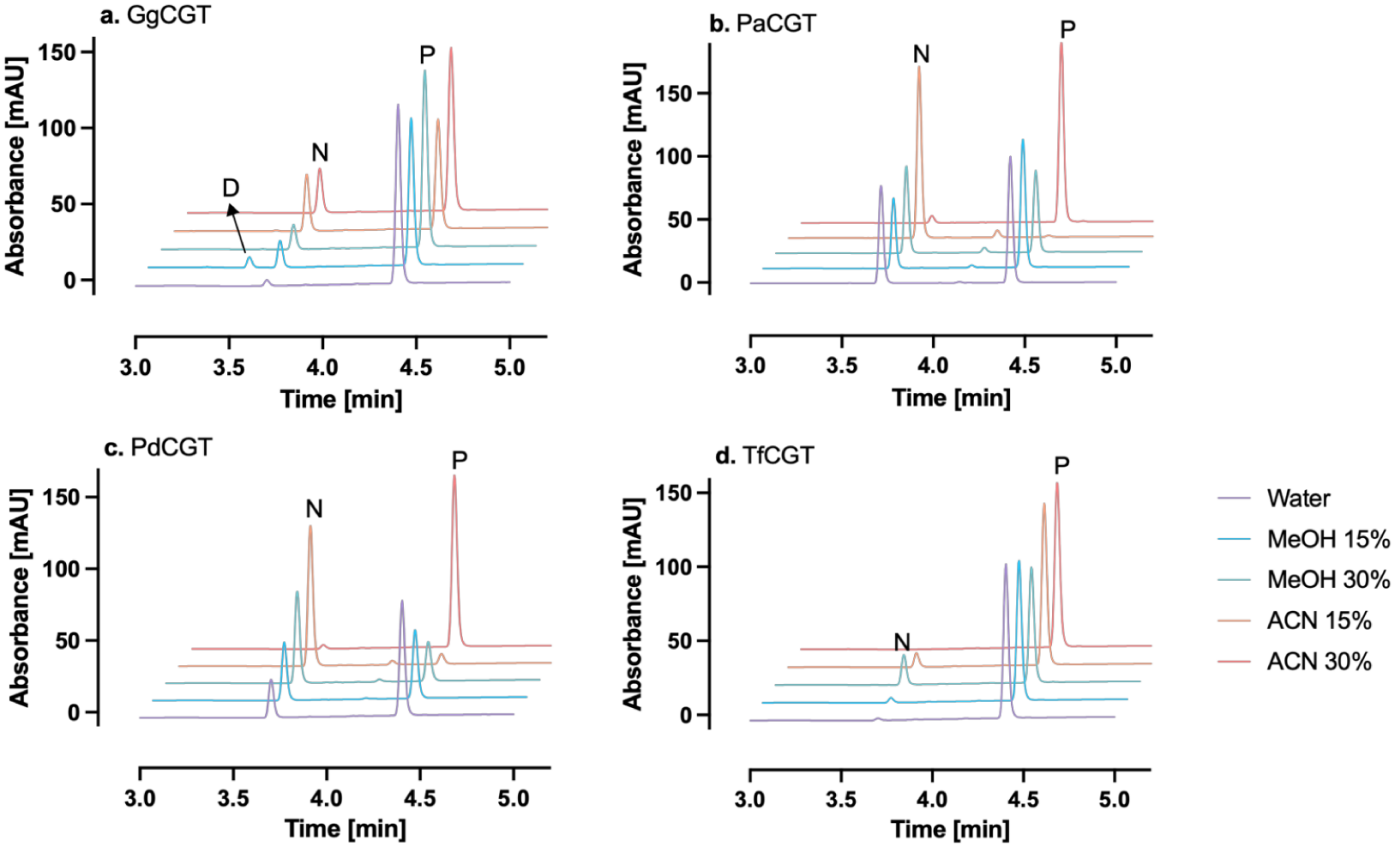
HPLC chromatograms showing the conversion of phloretin by halophytic *C*-GTs in reaction media with methanol or acetonitrile. Conversion in aqueous buffer (50 mM sodium phosphate, pH 8.0) was considered 100% for each enzyme. Reactions were run at 30 °C for 10 min and contained 5 μg/mL total protein and 50 μM phloretin. Please note that the enzyme load is 10% of what is used in Figure 2 to minimize enzyme consumption in our experiments. Conversion yields were calculated as the sum of nothofagin and phloretin 3′,5′-di-*C*-glucoside production.

To investigate if the observed preference for acetonitrile- or methanol-containing media over purely aqueous media is a specific property of halophytic *C*-GTs, we conducted the same study with three known *C*-GTs from nonhalophytic plants, namely, MiCGT from *Mangifera indica* (mango),^40^ FcCGT from *Fortunella crassifolia* (meiwa kumquat),^28^ and VaCGT from *Vigna angulariz* (adzuki bean)^41^ (Table 1). Some degree of increased conversion yields in organic solvents was also observed in this group, though not as prominent as for the halophytic *C*-GTs. For MiCGT (Figure 4a) and VaCGT (Figure 4c), maximum relative conversion yields were obtained in 15% methanol (v/v) (301% and 303%, respectively), while FcCGT gave maximum relative conversion in 30% methanol (v/v) (221%) (Figure 4b). Relative phloretin conversion yields for the halophytic and nonhalophytic *C*-GTs in aqueous buffers and organic solvents are given in Supporting Information 4.

**Figure 4:**
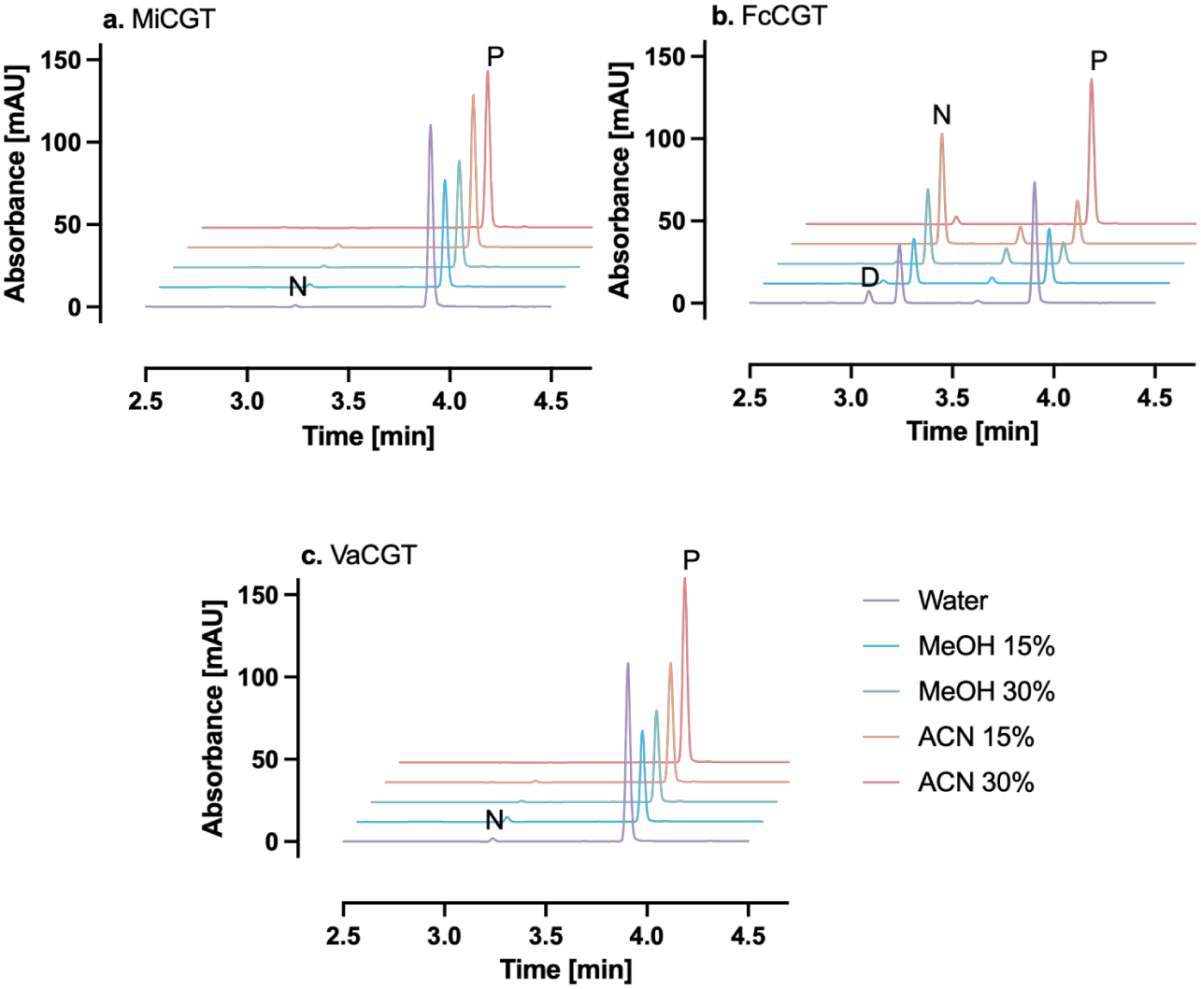
HPLC chromatograms showing the conversion of phloretin by nonhalophytic *C*-GTs in reaction media with methanol or acetonitrile. Conversion in an aqueous buffer (50 mM sodium phosphate, pH 8.0) was considered 100% for each enzyme. Reactions were run at 30 °C for 10 min and contained 5 μg/mL of total protein and 50 μM phloretin. Please note that the enzyme load is 10% of what is used in Figure 2 to minimize enzyme consumption in our experiments. Conversion yields were calculated as the sum of nothofagin and phloretin 3′,5′-di-*C*-glucoside production. The spectra are shifted ca. −0.5 min compared to previous HPLC results due to a slightly different elution protocol used on another HPLC instrument with the same model.

### 2.4 Temperature and pH Activity Profiles and Acceptor Stability Assessments

We further characterized the halophytic *C*-GTs by determining their temperature and pH activity profiles in aqueous buffers (no solvent). The temperature optima of halophytic *C*-GTs were observed to be in line with those of previously reported *C*-GTs, which range between 30 and 55 °C.^26–28,40–43^ No *C*-GT was active at 70 °C (Figure 5).

**Figure 5:**
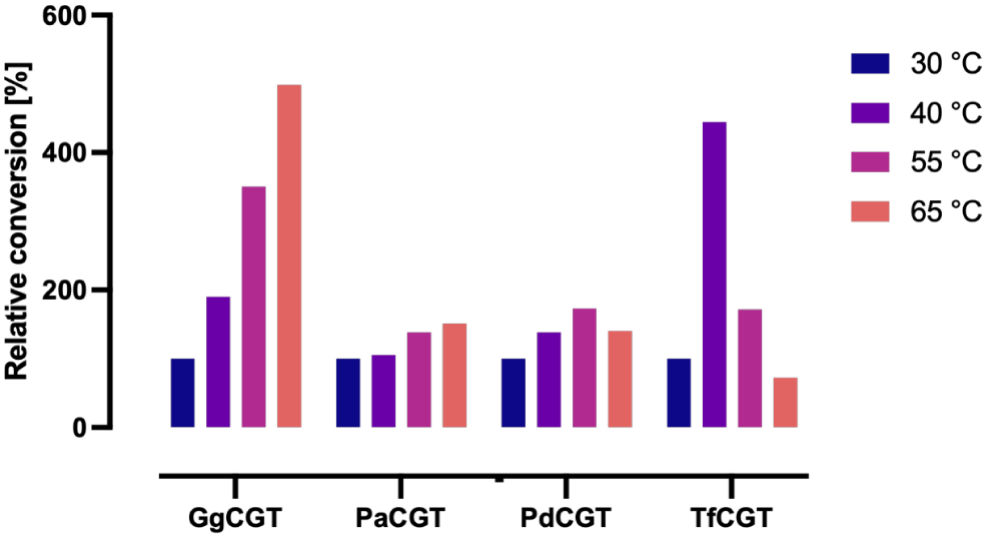
Relative phloretin conversion yields of halophytic *C*-GTs were determined at increasing temperatures. Conversion at 30 °C was considered 100% for each enzyme. Reactions were run for 10 min in aqueous buffer and contained 5 μg/mL total protein and 50 μM phloretin. Conversion yields were calculated as percentage emergence of nothofagin and phloretin 3′,5′-di-*C*-glucoside. No enzyme was active at 70 °C.

Next, we assessed the effect of pH on enzyme activity in four different buffer systems at 14 pH values (citric acid–trisodium citrate, pH 4.0–5.9; HEPES, pH 6.9–8.2; glycine–NaOH, 8.7–10.0; CAPS, pH 11.0–12.0). In line with many other GT1 enzymes,^27,43–45,46^ all halophytic *C*-GTs seemed to prefer alkaline conditions (pH 8.7–10.0, Figure 6). Both GgCGT and PaCGT showed maximum phloretin conversion at pH 9.2, while for PdCGT and TfCGT, it was at pH 10.0. Local maxima observed in citric acid–trisodium citrate buffer at pH 5.9 with GgCGT and PaCGT are probably due to switching to the HEPES buffer at pH 6.9. A sharp decrease in activity was observed for all enzymes at pH 11.0: Relative conversion yields of GgCGT and PaCGT dropped to 10.12% and 3.27%, respectively, while PdCGT and TfCGT experienced a complete loss of activity.

**Figure 6:**
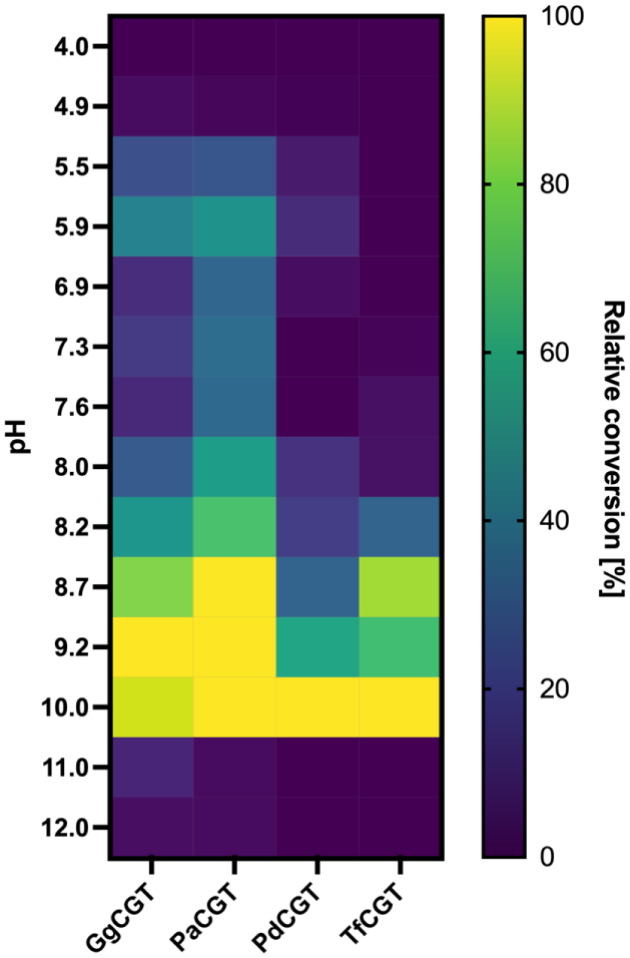
Heat map showing the pH range of phloretin conversion by halophytic *C*-GTs. The pH value at which the maximum conversion observed was considered to be 100% for each enzyme. Enzyme and phloretin concentrations were 2 μg/mL and 100 μM for GgCGT and PaCGT, respectively, and 5 μg/mL and 50 μM for PdCGT and TfCGT, respectively. Reactions were run at 40 °C for 10 min.

We also evaluated the activity of the enzymes at increasing phloretin and enzyme concentrations, since low chemostability toward aglycons and dilution-induced inactivation are often observed with plant GT1 enzymes, with aglycon concentrations as low as 50 μM causing more than 50% activity loss in several cases.^25^ GgCGT showed some degree of inhibition at low enzyme concentrations, with almost complete activity recovery (>99%) observed at 10 μg/mL of enzyme in the reactions (Figure 7a). PaCGT displayed remarkable chemostability under all conditions tested; the lowest conversion yield (94.3% ± 7.2%) was observed with 5 μg/mL enzyme at 50 μM phloretin with no apparent inhibition by enzyme dilution or substrate loading observed (Figure 7b) under the conditions tested. In contrast, PdCGT and TfCGT exhibited weaker chemostability profiles through different manners. PdCGT was found to be more susceptible to enzyme dilution rather than phloretin concentrations, since conversion showed only a modest drop at higher phloretin, but it scaled almost directly with enzyme loading, with almost full activity recovery (>99%) achieved with 40 μg/mL of enzyme (Figure 7c). TfCGT, on the other hand, was more susceptible to an increase in phloretin concentrations. The strong downturn proportional to substrate concentration outweighed any gain from enzyme loading, with only 58.0% ± 0.6% conversion observed at the highest enzyme-lowest substrate reaction (Figure 7d). The results obtained here are somewhat in line with their pH profiles as well (GgCGT and PaCGT retaining some activity over pH 11.0 while the remaining two lose activity completely).

**Figure 7:**
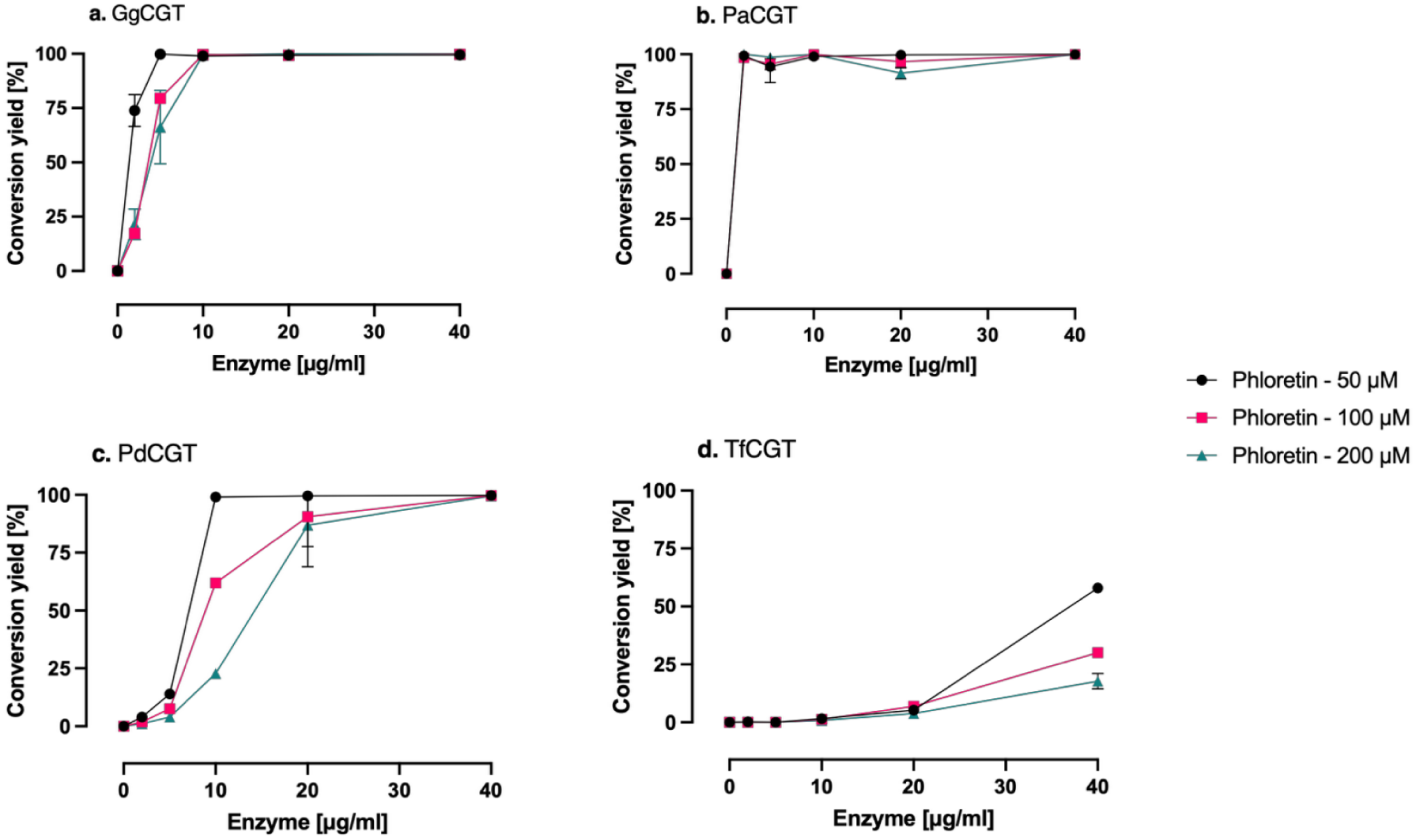
Effects of protein and phloretin concentrations on enzyme activities. Reactions were run at 40 °C for 1 h in an aqueous buffer (50 mM potassium phosphate, pH 8.0). Error bars represent the standard error of two replicates.

## 3 Discussion

Although the enzymatic glycosylation of small molecules via plant GT1 enzymes has been the subject of a great number of studies in the last decades, the poor solubility of the aglycons and the implications of this on the realization of feasible industrial-scale enzymatic glycosylation processes have been reflected upon sporadically. In a recent study, researchers included numerous organic solvents in reaction media to improve the solubility of 15-hydroxy cinmethylin for its glycosylation via UGT71E5 from *Carthamus tinctorius*; however, the enzyme was severely inhibited in the presence of all solvents tested.^47^ Efficient glycosylation of the substrate could be realized only after its complexation in 2-hydroxypropyl-β-cyclodextrin. The same strategy was applied for nothofagin production in a one-pot reaction with OsCGT coupled to a GmSuSy UDP-Glc regeneration system, resulting in 50 g/L final nothofagin concentration.^48^ Although not from the GT1 family, a bacterial cyclodextrin glycosyltransferase from the GH13 family of enzymes has been successfully used in a cosolvent system with DMSO to synthesize fisetin glycosides.^49^ An organic solvent-tolerant glycosyltransferase from *Bacillus licheniformis* PI15 was reported to efficiently glycosylate raspberry ketone in a reaction medium containing 10% DMSO, resulting in 26-fold higher conversion yield than that in an aqueous medium.^50,51^ To the best of our knowledge, no study exists in the literature assessing the potential of the plant GT1 family enzymes in organic solvents for enhanced glycosylation.

Our reactions in organic solvents contained only 5 μg/mL enzyme, in comparison to 50 μg/mL used during the initial glycosylation screenings. In the case of PaCGT, for example, almost 100% conversion to nothofagin could be achieved with either 50 μg/mL enzyme in aqueous medium (Figure 2c) or with 5 μg/mL enzyme in reaction medium containing 15% acetonitrile (v/v) (Figure 3d), without changing the phloretin concentration. Thus, perhaps the most important outcome of this study is that halophytic GT1 enzymes can achieve the same efficacy with much less protein in the reaction (9 times less in the case of PaCGT) with the use of organic solvents. This outcome has both economic and environmental implications, especially considering that industrial enzyme manufacturing processes relying on conventional feedstocks have high impacts on the environment.^52^ It would be interesting to investigate the effects of green solvents, such as bioethanol and tributyrate. We have also investigated the effects of methanol and acetonitrile on three nonhalophytic *C*-GTs, and although some degree of enhanced conversion was observed, the difference was not as prominent as that in halophytic *C*-GTs (Figure 4). Nevertheless, it is still not clear whether this difference is solely due to the halophytic origin of these enzymes, since the nonhalophytic *C*-GTs did not perform worse than PaCGT.

The adaptation mechanisms observed in salt-tolerant enzymes such as increased number of negatively charged residues on the protein surface^53^ or a reduction in overall hydrophobic interactions in the protein structure^54,55^ are well documented. However, we do not know if these mechanisms are relevant for the halophytic *C*-GTs evaluated in this study since, depending on the salt tolerance mechanisms of the plants, some of these enzymes might not even encounter high salt concentrations in their natural settings.

The optimum temperature and pH values of the investigated halophytic *C*-GTs correspond to those of previously reported ones, which range from 30 to 55 °C^26–28,40,42,43^ and pH 8.0–10.0.^27,43–46^ It should be noted that in contrast to our observations, a previous study^27^ showed maximum phloretin glycosylation activity with GgCGT between 37 and 50 °C with a sharp decrease at 60 °C. Determination of the melting temperature of the enzymes should provide more information on their thermal stability. This could not be carried out in this study due to the low amounts of enzymes purified. We also could not obtain kinetic data for phloretin due to substrate inhibition. The same issue was reported before for GgCGT.^27^

While phloretin has very low solubility in water ((3.20 ± 0.02) × 10^–4^ mol/kg) at room temperature (298.2 K), its solubility is over 2500 times higher in methanol (0.83 ± 0.03 mol/kg).^14^ In theory, employing halophytic GT1s in organic solvent media can thus push the maximum amount of aglycon that can be included in the glycosylation reactions drastically. However, the low chemostability of GT1s toward their acceptor substrates is an issue that needs to be overcome to realize such high-yield glycosylation processes.

In a recent work of ours, 15 out of 18 plant GT1 enzymes assessed were markedly inactivated to varying degrees by enzyme dilution and 50 to 400 μM of apigenin, resveratrol, or scopoletin.^25^ When we tested the dilution-induced inactivation phenomenon for halophytic *C*-GTs against increasing phloretin concentrations, PaCGT stood out as the most robust biocatalyst, with no significant inactivation observed under any enzyme–phloretin concentration combinations tested, while PdCGT and TfCGT were inhibited mainly by enzyme dilution and substrate loading, respectively. Although it was suggested that the large, solvent-exposed hydrophobic acceptor site of GT1 enzymes might be involved,^25^ an interesting mechanistic explanation for this phenomenon was published recently, where the authors showed that despite being a competitive inhibitor, β-carotene greatly alleviated the substrate inhibition issue for the tobacco glycosyltransferase *Nb*UGT72AY1.^56^ It was hypothesized that the asymmetric cooperativity of the substrates was the main reason for substrate inhibition through the predominant formation of a nonproductive conformer at high substrate concentrations, with β-carotene acting as a placeholder that allows UDP-Glc to bind without the conformational change caused by the aglycon. Further investigations with halophytic *C*-GT reactions in polar organic solvents and increasing substrate concentrations might prove to be useful in improving our understanding of this phenomenon.

## 4 Conclusion

In conclusion, we have identified three novel phloretin *C*-GTs from halophytic plant species and, as a result of a serendipitous discovery, reported that these enzymes display enhanced performances in the presence of polar organic solvents, allowing higher product yields with much less enzyme. We believe the results presented here can serve as a basis for the discovery of robust and industrially relevant GTs in future studies.

## 5 Materials and Methods

### 5.1 Chemicals

All chemicals used throughout the experiments except for phloretin and its two glucosides were from Sigma-Aldrich with the highest available purity versions. Phloretin was purchased from TargetMol Chemicals Inc. as part of their Polyphenolic Natural Product Library. Nothofagin and phloretin 3′,5′-di-*C*-glucoside standards were purchased from Wuhan ChemFaces Biochemical Co., Ltd. (Wuhan, PRC).

### 5.2 Sequence Mining for Halophytic *C*-GTs

To discover novel *C*-GTs from halophytes, five well-characterized *C*-GTs from *Citrus japonica* (BBA18062.1), *Glycyrrhiza glabra* (6L5S), *Oryza sativa* subsp. *indica* Kato (C3W7B0.1), *Mangifera indica* (7VAA), and *Trollius chinensis* (6JTD) were used as queries in BLAST (tblastn) searches limited to all taxonomic familia that contain halophytic plant species present in the eHALOPH database (https://ehaloph.uc.pt, access date: 25/08/2023). This was achieved via an Entrez query with all known familia that contain halophytic species (Supporting Information 1). The results were manually screened for known halophytes on the species level. Filtering criteria were as follows: >50% sequence identity with at least one of the query sequences, presence of the DPF (FL) and PSPG motifs, instability index <45, and at least 300 mM salt concentration tolerated by the origin species. The phylogenetic tree in Figure 1 was built using iTOL.^57^

### 5.3 Heterologous Protein Production and Purification

Chosen sequences were added to N-terminal 6xHis-tag and TEV cleavage sites with linkers (MGSSHHHHHHSSGENLYFQGSS-) and cloned into pET28a­(+) vectors between the NcoI (5′) and XhoI (3′) restriction sites by Biomatik LLC (Canada). One Shot BL21­(DE3) Chemically Competent *Escherichia coli* (ThermoFisher Scientific) cells were transformed with the expression vectors via heat shock application as instructed by the manufacturer. Transformed colonies were chosen on Luria–Bertani (LB) plates with 50 μg/mL kanamycin at the end of an overnight incubation at 37 °C. Single colonies were picked the next day and grown in 5 mL of LB medium with 50 μg/mL kanamycin at 37 °C and 200 rpm until the optical density at 600 nm (OD_600_) reached ca. 0.6. Cells were then aliquoted and stored in 15% (v/v) glycerol at −80 °C for further experiments. For heterologous protein production, precultures with 1% (v/v) inoculum in 7.5 mL of LB medium with 50 μg/mL kanamycin were incubated at 37 °C and 200 rpm until the OD_600_ reached ca. 0.6. Precultures were then transferred to the same medium of 500 mL in 2 L baffled Erlenmeyer flasks at an inoculation ratio of 1.5% (v/v). Antifoam 204 dissolved in sunflower oil was also added to media at 0.005% (v/v) final concentration. Cells were grown under the same incubation conditions until OD_600_ reached ca. 0.7, then protein expression was induced with the addition of 0.5 mM of isopropyl β-d-1-thiogalactopyranoside (IPTG), followed by overnight incubation at 20 °C and 200 rpm. Next day, the cells were pelleted via centrifugation at 4700 × *g* for 30 min at 4 °C, then resuspended in Buffer A (50 mM potassium phosphate dibasic, 300 mM NaCl, 20 mM imidazole, pH 7.5) supplemented with cOmplete, EDTA-free Protease Inhibitor Cocktail tablets (Roche), and 10 μg/mL DNase I. Cells were lysed via ultrasonication (30 s on/30 s off for 12 min in total at 60% nominal power setting, corresponding to an observed output power of ca. 22 W) on ice with a VCX-130 Ultrasonic Processor (Medline Scientific). The lysates were centrifuged at 14,000 × *g* for 55 min at 4 °C to separate the cell debris. Supernatants were filtered through 0.45 μm syringe filters before purification. Protein purification was performed with 1 mL of HisTrap FF columns (Cytiva) on an ÄKTA pure FPLC system (Cytiva) in a cold room at 10 °C. The column was equilibrated with 10 column volumes (CV) of Buffer A (composition given above) before sample application. The washing step after sample application was carried out with 20 CV of 94.9% of Buffer A + 5.1% of Buffer B (50 mM potassium phosphate dibasic, 300 mM NaCl, and 500 mM imidazole, pH 7.5). Fractions of 1 mL were eluted from the column with the following program: Linear increase from 0% to 40% (v/v) Buffer B for 20 CV, 100% Buffer B for 5 CV, and 100% Buffer A for 5 CV. The flow rate was 1 mL/min throughout the purification. Protein concentrations in the fractions were determined via absorbance readings at 280 nm on a NanoDrop 2000 instrument (Thermo Fisher Scientific). The presence of the target proteins was verified by SDS-PAGE analyses. NuPAGE 4 to 12%, Bis–Tris, 1.0–1.5 mm, Mini Protein Gels (Thermo Fisher Scientific) were stained with InstantBlue Coomassie Protein Stain (Abcam Ltd.). The protein ladder used was a PageRuler Prestained Protein Ladder (Thermo Fisher Scientific). SDS-PAGE gel images were analyzed via Image Lab Software (Bio-Rad) to quantify the purity of the protein fractions. Fractions with the target proteins were pooled and buffer-exchanged with Amicon Ultra-15 centrifugal filters with 50 kDa MWCO (Millipore) against a storage buffer (25 mM potassium phosphate dibasic and 150 mM NaCl, pH 7.5) three times. Retentates containing the proteins were aliquoted and flash frozen in liquid nitrogen to be stored at −80 °C for further experiments.

### 5.4 Glycosylation Screening

Phloretin was chosen as the model acceptor molecule due to the presence of numerous potential *C*- and *O*-glycosylation sites that it harbors. Reactions of 100 mL containing 50 μM phloretin, 5 mM UDP-Glc, 50 μg/mL enzyme, 50 mM sodium phosphate, and 50 mM NaCl (pH 8.0) were run at 30 °C and 300 rpm on a thermoshaker and quenched with an equal volume of 100% methanol after 10 min, 1 h, and overnight. Reaction media were centrifuged at 10,000 × *g* for 10 min before HPLC analyses.

### 5.5 Glycosylation in Organic Solvents

To test the effect of polar organic solvents on enzyme activity, glycosylation reaction media including either 15% (v/v) or 30% (v/v) acetonitrile or methanol were used. Differing from the glycosylation screening experiments, enzyme and UDP-Glc concentrations were reduced to 5 μg/mL and 1 μM, respectively. Reactions run at 30 °C and 300 rpm were stopped with an equal volume of 100% acetonitrile after 10 min and centrifuged at 10,000 × *g* for 10 min before HPLC analyses.

### 5.6 Biochemical Characterization of *C*-GTs

To determine the active pH range of *C*-GTs, reactions were run in three buffer systems at 12 different pH values, namely, citric acid–trisodium citrate buffer (pH 4.0–5.9), 4-(2-hydroxyethyl) piperazine-1-ethane-sulfonic acid (HEPES) buffer (6.9–8.2), glycine–NaOH buffer (8.7–10.0), and *N*-cyclohexyl-3-aminopropanesulfonic acid (CAPS) buffer (11.0–12.0). Enzyme and phloretin concentrations were 2 and 100 μM for GgCGT and PaCGT, and 5 and 50 μM for PdCGT and TfCGT, respectively. Each reaction mixture contained 1 mM UDP-Glc. Reactions of 100 mL in 96-well plates were run at 40 °C and 300 rpm for 10 min and then quenched with an equal volume of 100% acetonitrile (v/v). Plates were centrifuged at 4,700 × *g* for 20 min before HPLC analyses. Conversion yields were normalized separately for each enzyme, assuming that the highest conversion observed corresponds to 100% relative activity.

### 5.7 Thermal and Chemical Stability Assessments

Temperature optima of the enzymes were determined via running 100 μL of reactions in 200 μL PCR strip tubes on a thermocycler for 10 min at 30, 40, 55, 65, and 70 °C. Enzyme and phloretin concentrations were 5 μg/mL and 50 μM, respectively. Each reaction mixture contained 1 mM UDP-Glc, 50 mM sodium phosphate and 50 mM NaCl as buffer (pH 8.0). Results were normalized separately for each enzyme, assuming that the conversion at 30 °C corresponds to 100% relative activity.

Chemostability of the enzymes against the substrate (phloretin) was assessed by running reactions containing 0, 2, 5, 10, 20, or 40 μg/mL enzyme and 50, 100, or 200 μM phloretin. Each reaction contained 500 μM UDP-Glc, 50 mM sodium phosphate and 50 mM NaCl as buffer (pH 8.0). Reactions of 100 μL in 96-well plates were run for 1 h at 40 °C without agitation and stopped with an equal volume of 100% acetonitrile (v/v). Samples were centrifuged at 4,700 × *g* for 20 min before HPLC analyses. Results were normalized separately for each enzyme, assuming the point at which the highest conversion observed corresponds to 100% relative activity.

### 5.8 HPLC Analyses

End reactions were analyzed with reverse-phase HPLC in an Ultimate 3000 Series system (Thermo Fisher Scientific) using a Kinetex C18 column (2.6 mm, 100 Å, 100 × 4.6 mm, Phenomenex) at 30 °C. The mobile phase was water and acetonitrile containing 0.1% formic acid. The injection volume was 10 μL. The following gradients were employed during analysis: From 5% to 25% acetonitrile (v/v) in 1.5 min, from 25% to 80% acetonitrile (v/v) in 2 min, from 80% to 100% acetonitrile (v/v) in 1.5 min, 100% acetonitrile (v/v) for 1 min, from 100% to 5% acetonitrile (v/v) in 30 s, and 5% acetonitrile (v/v) for 1.5 min. The flow rate of the mobile phase was kept constant at 1 mL/min. Absorbances at 290 nm were recorded with a UV detector. Data were analyzed via Chromeleon 7 software (Thermo Fisher Scientific). Conversion yields were calculated as the percentage emergence of nothofagin and phloretin 3′,5′-di-*C*-glucoside.

### 5.9 Statistical Analyses and Data

Data in Figures 2–6 are presented as single measurements due to the limited amounts of soluble enzyme obtained. Data in Figure 7 are presented as duplicate measurements whenever possible, with error bars representing the standard error.

## Supplementary Material



## Notes

Supporting Information 1. List of taxonomic families included in the tblastn search as Entrez query for the discovery of halophytic *C*-GT sequences. Supporting Information 2. SDS-PAGE gel image of buffer-exchanged protein fractions. Supporting Information 3. HPLC calibration curves for phloretin, nothofagin, and phloretin 3′,5′-di-*C*-glucoside. Supporting Information 4. Relative phloretin conversion yields of nonhalophytic (MiCGT, FcCGT, VaCGT) and halophytic *C*-GTs (GgCGT, PaCGT, PdCGT, TfCGT) (PDF)

## #

Associate Professorship of Biotechnology of Natural Products, Technical University of Munich, Liesel-Beckmann-Str. 1 85354 Freising, Germany

## Abbreviations

ACN: acetonitrile
CAPS: *N*-cyclohexyl-3-aminopropanesulfonic acid
CAZy: carbohydrate-active enzymes
*C*-GT: C-glycosyltransferase
CV: column volumes
DMSO: dimethyl sulfoxide
EDTA: ethylenediaminetetraacetic acid
FPLC: fast protein liquid chromatography
GT1: Glycosyltransferase Family 1
HEPES: 4-(2-hydroxyethyl)­piperazine-1-ethane-sulfonic acid
HPLC: high pressure liquid chromatography
IPTG: isopropyl ß-d-1-thiogalactopyranoside
LB: Luria–Bertani
RT: retention time
MeOH: methanol
SDS-PAGE: sodium dodecyl sulfate–polyacrylamide gel electrophoresis
UDP-Glc: uridine diphosphate glucose
UGT: uridine diphosphate-dependent glycosyltransferase
