# Supplementary material for: Halophytic C‑Glycosyltransferases Enable C‑Glycosylation in Organic Solvents
Source: ACS Omega. 2025 Nov 16;10(46):55909–19. doi: 10.1021/acsomega.5c07452 (PMC12658608; doi:10.1021/acsomega.5c07452)
Supplement: Supplementary file 1 [file ao5c07452_si_001.pdf]

Halophytic *C*-glycosyltransferases enable *C*-glycosylation in organic solvents

Onur Kırtel[a*], Lea Helena Strother[a], Natalia Putkaradze[a*], Ditte Hededam Welner[a*]

[a] *The Novo Nordisk Foundation Center for Biosustainability, Technical University of Denmark, Søltofts Plads 220, DK-2800, Lyngby, Denmark*

**Current address: Associate Professorship of Biotechnology of Natural Products, Technical University of Munich, Liesel-Beckmann-Str. 1 85354 Freising, Germany*

Email addresses:

Onur Kırtel (okirtel@dtu.dk) *(*Co-corresponding author*)

Lea Helena Strother (least@dtu.dk)

Natalia Putkaradze (natalia.putkaradze@tum.de)

Ditte Hededam Welner (diwel@dtu.dk) *(*Co-corresponding author*)

Keywords

Halophyte, extremophile, glycosylation, *C*-glycosyltransferase, glycosyltransferase family 1, phloretin, natural products

Conflicts of Interest: None

Acanthaceae, Aizoaceae, Alismataceae, Amaranthaceae, Anacardiaceae, Annonaceae, Apiaceae, Apocynaceae, Arecaceae, Asparagaceae, Asphodelaceae, Asteraceae, Basellaceae, Bataceae, Bignoniaceae, Boraginaceae, Brassicaceae, Caryophyllaceae, Casuarinaceae, Celastraceae, Cleomaceae, Clusiaceae, Combretaceae, Convolvulaceae, Cymodoceaceae, Cynomoriaceae, Cyperaceae, Didiereaceae, Elaeagnaceae, Euphorbiaceae, Fabaceae, Frankeniaceae, Gentianaceae, Goodeniaceae, Hydrocharitaceae, Iridaceae, Juncaceae, Juncaginaceae, Lamiaceae, Lecythidaceae, Linaceae, Lythraceae, Meliaceae, Myoporaceae, Myristicaceae, Myrtaceae, Nyctaginaceae, Oleaceae, Onagraceae, Orobanchaceae, Pandanaceae, Papervaceae, Phrymaceae, Plantaginaceae, Plumbaginaceae, Poaceae, Podocarpaceae, Polygonaceae, Portulacaceae, Posidoniaceae, Potamogetonaceae, Primulaceae, Pteridaceae, Putranjivaceae, Resedaceae, Restionaceae, Rhamnaceae, Rhizophoraceae, Rubiaceae, Ruppiaceae, Rutaceae, Salicaceae, Salvadoraceae, Sapindaceae, Sarcobataceae, Saururaceae, Scrophulariaceae, Solanaceae, Sonneratiaceae, Sterculiaceae, Surianaceae, Tamaricaceae, Tetrameristaceae, Typhaceae, Ulmaceae, Verbenaceae, Xanthorrhoeaceae, Zosteraceae, Zygophyllaceae

Supporting Information 1. List of taxonomic families included in the tblastn search as Entrez query for the discovery of halophytic *C*-GT sequences.

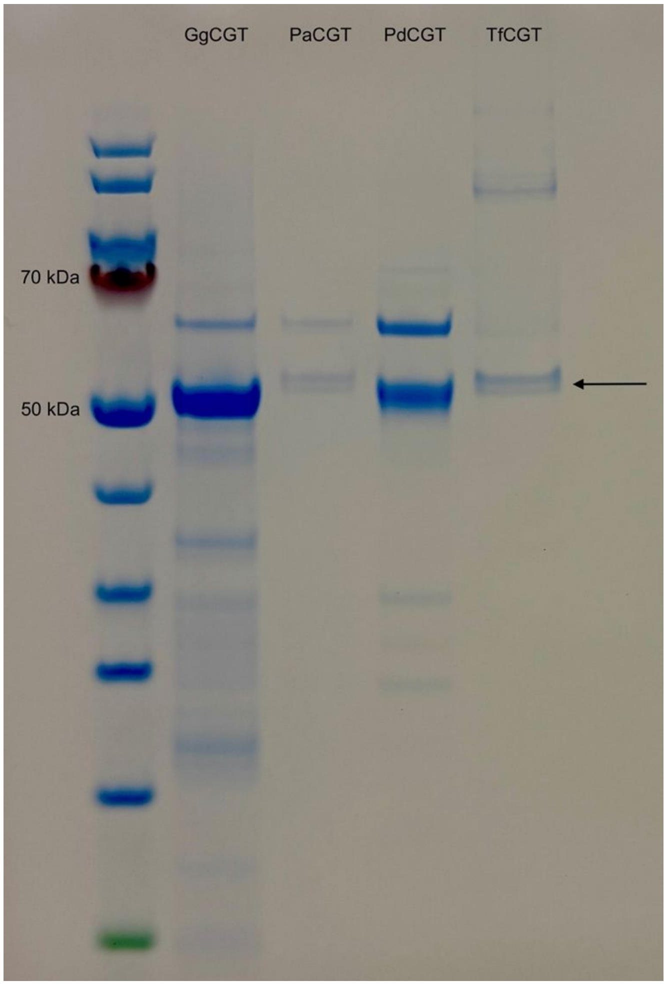

Supporting Information 2. SDS-PAGE gel image of buffer-exchanged protein fractions. Apparent molecular weights of the bands in the protein ladder were determined according to the information provided by the manufacturer in relation to the gel and buffer system used. The arrow indicates where the target proteins appear. Image Lab Software (Bio-Rad) was used to quantify the purity of each target protein, while protein concentrations were determined via Nanodrop measurements and were as followed: GgCGT: 1.872 mg/mL, 57.5% purity; PaCGT: 0.122 mg/mL, 36.0%; PdCGT: 0.912 mg/mL, 56.6% purity; TfCGT: 0.158 mg/mL, 40.5% purity.

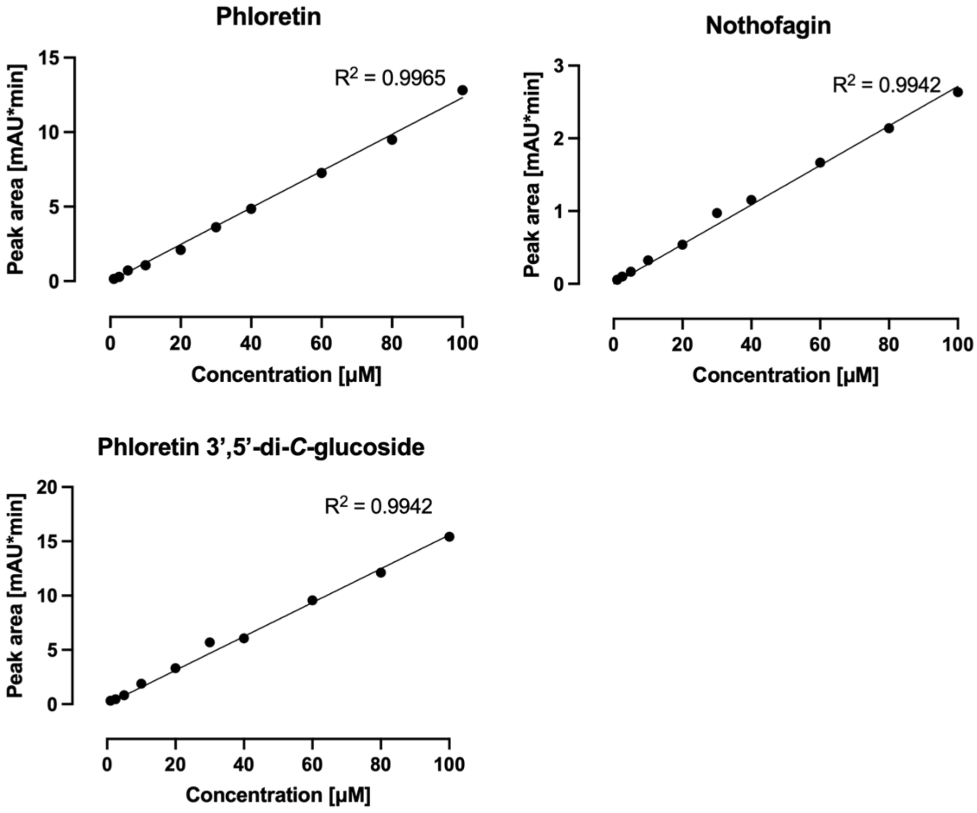

Supporting Information 3. HPLC calibration curves for phloretin, nothofagin and phloretin 3’,5’-di-*C*-glucoside. The HPLC method used was the same as described in Materials & Methods. Data were fit to linear functions by least squares with respective R^2 values given on each graph.

Enzyme / Buffer	Water	MeOH 15%	MeOH 30%	ACN 15%	ACN 30%
MiCGT	100.00	301.15	181.61	225.29	21.84
FcCGT	100.00	132.00	221.50	201.96	12.76
VaCGT	100.00	302.55	92.36	70.06	0.00
GgCGT	100.00	629.25	362.59	1095.24	664.63
PaCGT	100.00	81.67	119.71	227.68	8.48
PdCGT	100.00	193.13	302.99	412.21	1.54
TfCGT	100.00	245.31	1562.50	600.78	0.00

Supporting Information 4. Relative phloretin conversion yields of non-halophytic (MiCGT, FcCGT, VaCGT) and halophytic *C*-GTs (GgCGT, PaCGT, PdCGT, TfCGT).
